# Oxygen Monitoring Equipment for Sewage-Sludge Composting and Its Application to Aeration Optimization

**DOI:** 10.3390/s18114017

**Published:** 2018-11-18

**Authors:** Guodi Zheng, Yuewei Wang, Xiankai Wang, Junxing Yang, Tongbin Chen

**Affiliations:** 1 Center for Environmental Remediation, Institute of Geographic Sciences and Natural Resources Research, Chinese Academy of Sciences, Beijing 100101, China; wangyw.16s@igsnrr.ac.cn (Y.W.); wangxk.14s@igsnrr.ac.cn (X.W.); yangajx@igsnrr.ac.cn (J.Y.); chentb@igsnrr.ac.cn (T.C.); 2 College of Resources and Environment, University of Chinese Academy of Sciences, Beijing 100049, China

**Keywords:** compost, oxygen concentration, detection, monitor, aeration optimization

## Abstract

Oxygen is an important parameter for organic-waste composting, and continuous control of the oxygen in a composting pile may be beneficial. The oxygen consumption rate can be used to measure the degree of biological oxidation and decomposition of organic matter. However, without having a real-time online device to monitor oxygen levels in the composting pile, the adjustment and optimization of the composting process cannot be directly implemented. In the present study, we researched and developed such a system, and then tested its stability, reliability, and characteristics. The test results showed that the equipment was accurate and stable, and produced good responses with good repeatability. The equilibrium time required to detect oxygen concentration in the composting pile was 50 s, and the response time for oxygen detection was less than 2 s. The equipment could monitor oxygen concentration online and in real time to optimize the aeration strategy for the compost depending on the concentration indicated by the oxygen-measuring equipment.

## 1. Introduction

Composting is an important method for the treatment of organic waste, such as sewage sludge, livestock manure, and food waste [1]. Oxygen plays a key role in composting and is used to judge whether or not the process is complete [2,3]. To optimize biological activity in the composting process, different control strategies for the supplementing of oxygen have been studied. In general, temperature and oxygen content are considered as the key parameters in assessing microbial activity [4]. The decomposition of organic waste is divided into aerobic and anaerobic decomposition, with aerobic decomposition occurring at a faster rate [5,6].

Microbial activity is the determinant of temperature changes in compost [7,8]. Micro-organisms decompose organic matter and simultaneously release heat, thus increasing the temperature of the compost pile. As microbial activity decreases or stops, the temperature of the pile cannot increase, and can even decrease under the influence of other factors. The concentration of oxygen influences the micro-organism species that are present and the species microbial activity, therefore oxygen is an important influence on the temperature of the pile. Miyatake and Iwabuchi found that compost temperature rises quickly when oxygen levels are sufficient; otherwise, the temperature rises slowly [9]. Aside from the heating rate, the authors also found that the peak temperature of the studied compost pile was 70 °C at an oxygen concentration of 20.9%, in contrast to 60 °C at an oxygen concentration of 3.16% (*v*/*v*). Different temperature conditions affect the type (*v*/*v*) and the activity levels of micro-organisms, thereby affecting the maturity and decomposition of the organic matter. When oxygen supply is insufficient, organic degradation is reduced [5]. A sufficient oxygen supply can accelerate the stability and maturity of compost, whilst an insufficient supply may cause compost instability and biological toxicity [5,10,11]. Sterilization is one of the main tasks of aerobic composting, and temperature has an important effect on the inactivation of pathogens. The suitable temperature range for the survival of most pathogens is 45–60 °C, and the life activities of the bacteria under overheating are inhibited [12]. When oxygen content is insufficient or microbial activity is weakened, the rate of rising temperature decreases, the peak temperature lowers, and the inactivation effect on pathogens is decreased. The oxygen supply rate also affects the conversion of nitrogen. A high oxygen supply rate increases the NH_4_^+^–N content and reduces the NO_x_^−^–N content of the compost, which changes the compost’s stability [13]. Frederick et al. found that composting properties are unstable under a low oxygen supply and are stable under a high oxygen supply [5]. Unstable compost products also inhibit seed germination and plant growth [14].

Turning and forced aeration can improve the microenvironment of the compost pile and thereby supply the oxygen needed for microbial activity [15], thus effectively controlling the body temperature [3]. Usually, aeration by mechanical means is needed to keep oxygen concentration in the pile at a certain level to accelerate composting. Michel et al. reported that the best oxygen-consumption rate for garden waste compost was 2.1 mL/min, and a stable composting product can be achieved after 40 days [16]. The presence of oxygen in the composting material can also affect odor generation [17]. High levels of oxygen concentration decrease the emission rates and cumulative amounts of ammonia, carbonyl sulfide, carbon disulfide, and methlymercaptan during composting; wherein methlymercaptan was even extinguished in the subsequent stage [18]. In contrast, volatile organic acid (VOA) and greenhouse gas (GHG) can be generated with an inadequate oxygen level, but not when the oxygen is abundant [5,19]. Shen et al. also found that odors can be emitted from sewage-sludge composting piles when the oxygen concentration is below optimal [20].

The oxygen-consumption rate is used to measure the degree of biological oxidation and the decomposition of organic matter. Compost maturity can be judged by changes in the pile’s respiration, measured in terms of oxygen consumption and the intensity of carbon dioxide generation [2]. Through detecting the oxygen or carbon dioxide concentration in the exhaust, the oxygen concentration and consumption rate can be determined for the mechanized compost production system [21,22,23,24,25].

Wiley first reported on the respiration of a composting pile. The author used the quantity of carbon dioxide as an index for measuring the respiration indicators [26]. Epstein reported that compost was stable at a carbon dioxide emission level <5 mL(C)/g (compost carbon) and mature <2 mL(C)/g (compost carbon) [27]. Pressel detected the oxygen consumption of produced compost after composting for 0, 10, 14, and 20 days, as well as 8 months [28]. The results showed that the order of oxygen consumption was 0 days > 14 days > 10 days > 20 days > 8 months, and the oxygen consumption of the compost after 8 months was much less than at the other time points. This means that oxygen consumption decreases as compost stability increases.

William studied compost stability by measuring respiration with aerobic respirometry [29]. The author found that the rate of oxygen consumption can reflect on compost stability because compost with good stability consumes little oxygen. Therefore, a biological respiration parameter other than the physical and chemical parameters was found to indicate composting stability. Lasaridi also found that the respiration rate was a stable parameter for measuring the composting stability of sewage-sludge, municipal-solid-waste, and pulp-sludge composting [2]. At the beginning of the composting process, the oxygen-consumption rate of these three compost types was 19.6, 18.9, and 6.6 mgO_2_/g VS/hr, respectively. At the end of the composting (after two months), all of the oxygen consumption rates were lower than 2.5 mgO_2_/g VS/hr. Even when the composting time proceeded beyond two months, the oxygen-consumption rates changed minimally. Seed-germination testing also showed that the compost was stable after two months of composting. This work quantitatively proved that the oxygen consumption rate can be used to judge compost stability.

Most previous studies about the effect of oxygen on compost stability have focused on the relation of the respiration rate with the compost-material stability. However, this required the sampling of the material and the removal of the sample to a laboratory to measure the oxygen consumption using an oxygen sensor [2,14,16,28,29]. The lack of field detection capability is an obvious limitation of this method of oxygen measurement, because current methods cannot monitor the process in real time, and the result is only a simulation of the composting process. Oxygen concentration is dynamic and undergoes rapid continuous change. It varies between the different stages of composting or even within the same stage at a different period after aeration. Oxygen concentration changes very quickly, and it is exhausted a few minutes after aeration. Thus, the measurement of oxygen concentration requires a responsive measuring device. That is, only when the continuous automatic monitoring of oxygen is realized could meaningful studies of the composting process be achieved.

Currently, oxygen measurement is more focused on gas and liquid media through mature methods, such as the measurement of oxygen in air, mines, tunnels, and automobile exhausts, as well as the oxygen in water and liquid steel. Electrochemical oxygen sensors are used when the measuring object is at a normal temperature [30]. The measurement principle is that oxygen directly reacts with the electrolyte in the sensor when it comes into contact with the sensor’s electrode. However, this kind of sensor cannot be used in a high-temperature environment, even though it has a high response rate and measurement precision. Zirconia oxygen sensors are often used in high-temperature environments, such as automobile exhausts or flue gas from boilers [31]. The basic principle involves the use of zirconia as a solid electrolyte material to conduct oxygen ions and generate electromotive force. However, these sensors are large and need to be heated.

Continuous oxygen control of a compost pile would be greatly beneficial in allowing aeration changes to be made online and in real time. This would help avoid anaerobic conditions, which is very important when aiming to increase the temperature of a pile and it would also allow the direct adjustment and optimization of the composting process [14]. In the past, some control composting technologies determined oxygen concentration outside the pile via oxygen feedback. There is also some equipment that could detect oxygen concentration in a composting pile, such as a new automatic composting controller that has been developed using the oxygen uptake rate measure as the measured variable [4]. However, moisture in the air of a composting pile is high, and the temperature of the pile is changeable. To eliminate the effects of moisture and temperature difference on the measurement accuracy, this study aimed to research and develop oxygen-measurement equipment that could pump the gas in the compost pile and determine the oxygen concentration. This study’s goal was to research and develop real-time, online oxygen-measurement equipment, and to study the changes in oxygen concentration and expenditure, as well as the spatial characteristics of a compost pile during different stages of the composting process. Based on the above goals, we could analyze the relationship between oxygen supply and temperature change to optimize the composting process.

## 2. Material and Methods

### 2.1. Design Requirements

In order to accurately measure oxygen concentration in composting material, and to provide guidance to the process control for industrial production, it was determined that the oxygen-monitoring system should meet the following requirements: (1) High measurement accuracy; (2) quick response with a short equilibrium time; (3) stable measurement results with little data drift; (4) reproducible data; and (5) continuous automatic monitoring. For the study and autocontrol of the composting process, it was most important that the oxygen-monitoring system could operate automatically online and in real time.

### 2.2. Measuring Principles

**Figure 1:**
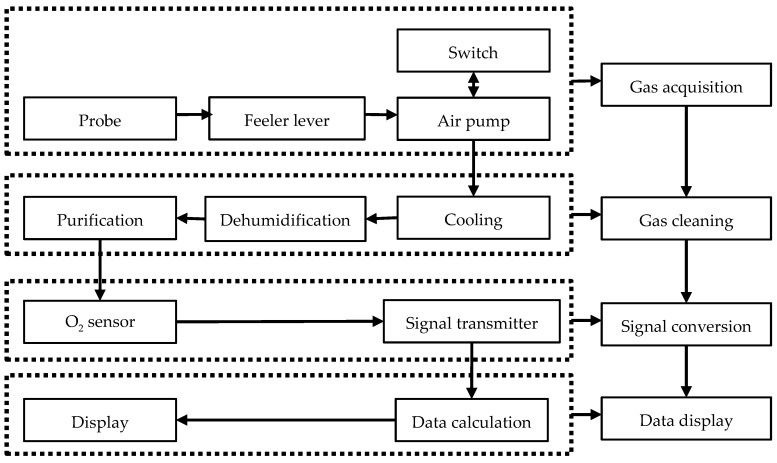
Schematic diagram of the equipment structure.

The oxygen-monitoring equipment included gas-acquisition, gas-cleaning, signal-conversion, and data-display systems (Figure 1). The gas-acquisition system, which included a probe, feeler lever, gas circuit, air-pump units, and an autocontrol switch, was designed to detect oxygen in the solid medium. To avoid the interference of high temperatures and foreign gases, the gas-cleaning system included gas-cooling, dehumidification, and purification units. The signal-conversion system included oxygen-sensor and signal-transmitter units, as well as a conveyor line. The data display and output system included data-calculation, display, and output units. The total volume of the gas-acquisition system was less than 2 mL, and the sensitivity of the equipment was ensured for its short equilibrium time. Electrical signals were produced and converted into digital signals as extracted gas flows through the oxygen sensor. The oxygen-concentration signals were then sent in real time to the data-acquisition card through a transmission line and the signals were then stored in a computer.

Usually, the sensitivity of the sensors is affected by humidity, temperature, carbon dioxide, and other factors. The most important factor is humidity, because the sensor could be damaged if water comes into contact with it. To increase the sensitivity, we designed a gas-cleaning system (Figure 1). The temperature and humidity of the gas pumped from the compost pile were lowered and cleaned before the gas contacted the sensors. The oxygen sensor used the Winsen ME2-O2 (Winsen Electronics Technology Co., Ltd., Zhengzhou, China). The signal transmitter was a digital to analog converter TM7705 (Reference voltage: 5 V, Highdo Co., Ltd., Wenzhou, China). The principle of oxygen detection in all oxygen detectors is the same, but the difference between the detectors is the gas cleaning system. To eliminate the influence of moisture and temperature difference, as well as other factors, in the measurement accuracy, the air pumped from the pile was measured by the process of: lowering temperature, dehumidify, and clean. The cooling treatment of the air was the immersion of the gas pipeline in a 20 °C water bath. Then the air was passed through a desiccant which was soda-lime. Finally, the air passed through an activated carbon filter.

### 2.3. Equipment Reliability Test

Different oxygen concentrations were attained by mixing high-purity nitrogen and oxygen in different proportions. The oxygen concentrations of the mixing gases were 0, 1%, 3%, 5%, 9%, 13%, 17%, and 21%. Then, the oxygen concentration was measured using the prototype measurement equipment. All the analyses were carried out in triplicate to reduce the error margin. Then, the determined and actual values of oxygen concentration were compared. The atmospheric oxygen level was also detected to check the precision, response time, and equilibrium time of the instrument.

Sewage sludge was collected from the Qinhuangdao Sludge Treatment Plant in Hebei Province, China. The water content of the sludge was approximately 80% after mechanical dewatering. Sawdust was obtained from a local timber-processing plant, where the diameter of the sawdust was approximately 1.0 cm, and the moisture content was 4.0–5.0%. Based on the moisture content and the C/N ratio of the sludge (water content, 80%) and sawdust (water content, 13.7%), the compost material was adjusted to a sludge/sawdust ratio of 6:1 (*v*/*v*). The compost experiment was conducted in a full-scale plant with a processing capacity of 200 t·d^−1^. The plant possessed 21 fermentation tanks, and the volume of each fermentation tank was 200 t. The compost pile size was 35 × 5 × 2.2 m. Intermittent aeration was performed for sewage-sludge composting in the plant. The aeration system was compatible with the computer controls for air volume. The oxygen-sensor system was placed on the top of the composting pile, and probes were placed 1.2 m beneath the surface of the pile. The composting period was 21 days. Oxygen concentration in the pile was measured in real time and online. To analyze the performance of the monitoring equipment during the composting process, the oxygen concentration was detected for a single aeration cycle.

## 3. Results and Discussion

### 3.1. Equipment Stability

**Figure 2:**
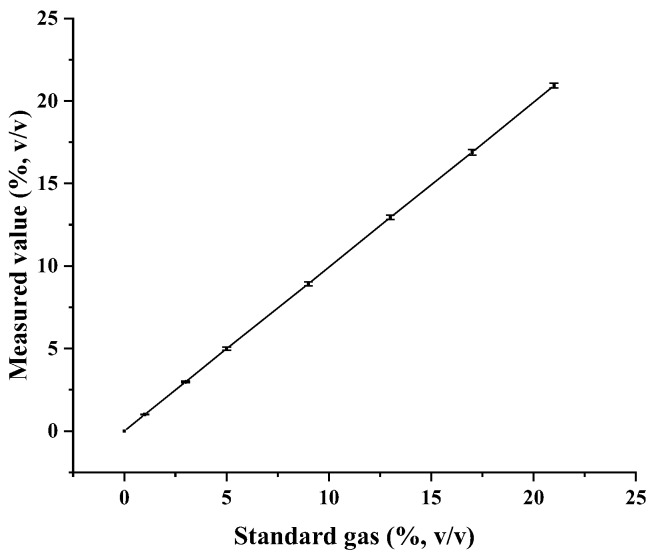
Oxygen concentrations of the mixed gases with high-purity nitrogen and oxygen in different proportions for equipment stability testing.

Oxygen-monitoring equipment was used to measure the oxygen concentration which was then compared to the actual, known value in the mixed gas. Good agreement and significant correlation were shown between the concentrations (Figure 2). The oxygen-monitoring equipment demonstrated excellent stability, good measurement accuracy, and reproducibility.

Composting is a complex biochemical process, wherein the components of produced gases are complicated, and predominantly include ammonia, hydrogen sulfide, and water, as well as mercaptan and thioether [32]. To ensure the measurement accuracy of the oxygen-monitoring equipment, it was placed in a gas environment with ammonia, hydrogen sulfide, and water. The results showed that the response time of the equipment was less than 2 s, and that the stability of the equipment was good.

**Figure 3:**
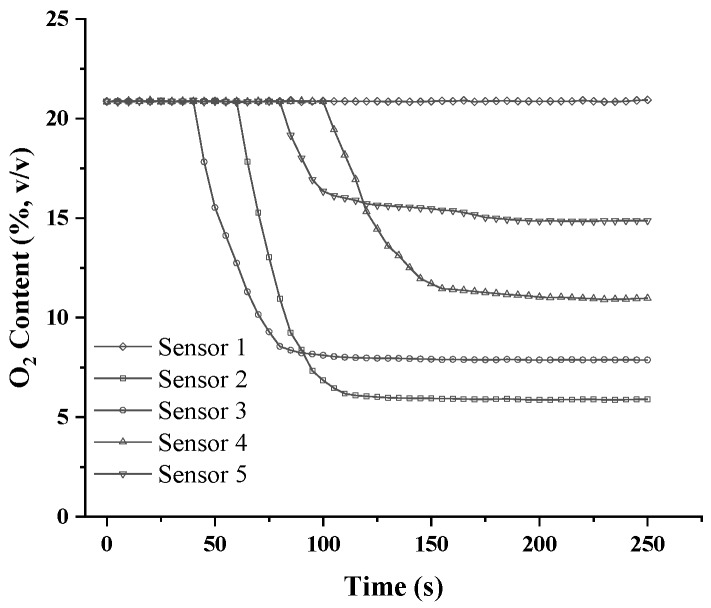
Oxygen concentrations in air, and the equilibrium time of the oxygen-concentration monitoring equipment in the composting pile.

In order to check the reliability of the equipment to measure oxygen, the gas-acquisition system was placed in air, and the air pump and acquisition system were switched on. A datapoint of the measured oxygen concentration was acquired every 5 s. The measurement period lasted 250 s. Statistical analysis was performed on 300 datapoints (SD 0.04), and the results were shown to be stable and accurate (Figure 3). The resolution of the sensor was 0.02% (*v*/*v*), and the limit of detection was 0~21%. For the true-accuracy composting requirement, the sensor-detection resolution and limit were sufficient, and it could be used for composting to optimize the aeration strategy.

Usually, the aeration cycle of a composting pile is more than 20 min, and it can be as long as 60 min. Therefore, the response time and reliability of the equipment met the requirements of oxygen detection in composting.

### 3.2. Equipment Reliability

**Figure 4:**
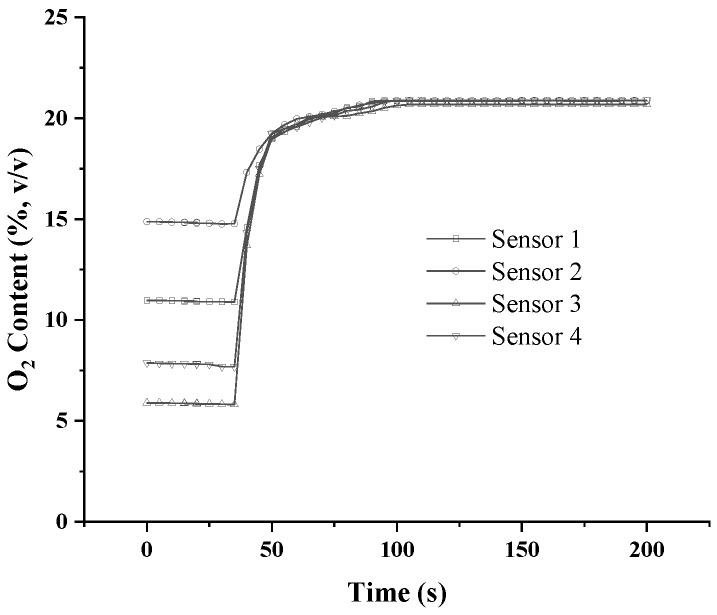
Equilibrium time of the oxygen-concentration monitoring equipment in air.

To check the equilibrium time of the oxygen-monitoring equipment, it was successively tested in compost and in air. The probes were placed at 1.5, 1.2, 0.9, and 0.6 m below the surface. Although oxygen concentrations varied for the probes that were placed at different depths, the equilibrium time was 50 s (95% of the potential value reached) for each piece of the oxygen-monitoring equipment (Figure 3). When the probes were placed in air, the equilibrium time was 50 s, and all the oxygen-concentration values were equal to those measured in air (Figure 4).

### 3.3. Equipment Characteristics

The equipment was proposed to monitor the oxygen-concentration online and in real time without requiring a significant amount of sampling or experimental work in the laboratory. The equipment allowed for continuous automonitoring and storage. The conditions of the composting pile were directly reflected in the data and it could be used to determine whether or not the aeration of the pile should be modified, for example, if it should be decreased to avoid unnecessary energy waste and to reduce the energy consumption of composting. The equipment also afforded a new method to study composting, as it could monitor changes in the oxygen concentration at intervals of several seconds.

Thus, the relationship between the oxygen-consumption rate and compost-maturity parameters, such as temperature, rate of emergence, ammonia, and NO_3_^−^, could be determined. Microbial life activity and community succession could also be studied in greater depth, and methods for cultivating and stimulating microbial decomposition activities to promote pile temperature increase by guaranteeing oxygen supply in the mesophilic phase could be formulated. At the same time, heat loss could be avoided by reducing aeration when oxygen supply is sufficient. To maintain microbial life activities, oxygen supply should be modified in the thermophilic phase to enhance organic matter biodegradation.

### 3.4. Application of the Equipment to Sludge Compost

**Figure 5:**
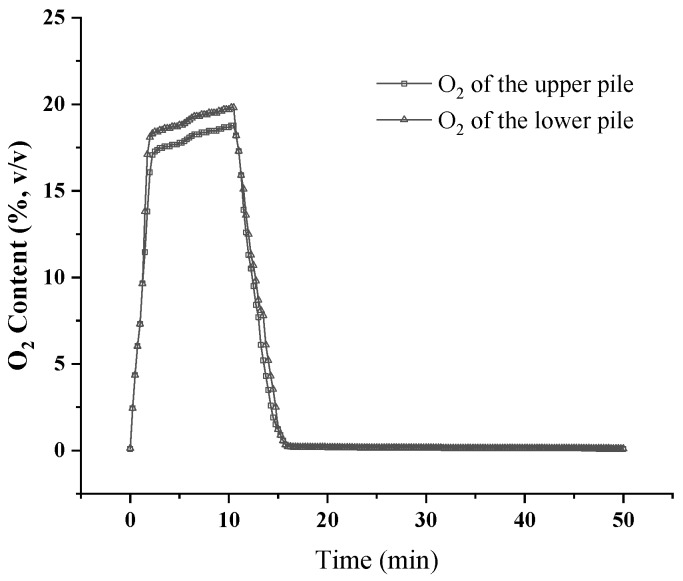
Oxygen concentrations of the composting pile in an aeration cycle.

In a single aeration cycle of the composting process, oxygen is consumed for microbial activity and new oxygen can then be supplied by aeration (Figure 5). In the current study, the fan was turned off after 10 min of aeration in a 50 min cycle. After aeration began, the oxygen concentration rapidly increased, although it became very low as time progressed. Oxygen concentrations in the pile increased to 16.1% (*v*/*v*, upper pile) and 18.1% (*v*/*v*, lower pile) within 2 min, and the concentrations further increased to 18.1% (*v*/*v*, upper pile) and 19.2% (*v*/*v*, lower pile) within 6 min. At the end of the aeration period, the oxygen concentration of the upper pile had increased to 18.8% (*v*/*v*), in contrast to the 19.8% (*v*/*v*) of the lower pile.

There is currently no oxygen-measurement equipment that can detect the oxygen concentration in a composting pile. Using this equipment, the minimum and maximum oxygen concentrations of the pile can be detected. The time taken for the concentration to reach its peak and the rate of rise and fall can be calculated through real-time online monitoring. From Figure 5, we can see that oxygen concentration in the pile increased to its highest concentration in 6 min, and no further substantial increase occurred in the remaining part of the aeration time (4 min). After the end of the aeration period, the oxygen concentration in the reactor was consumed to a large extent within 5 min and it reached a minimum. Therefore, the aeration strategy of the compost could be optimized depending on the oxygen concentration of the pile. For example, the aeration time could be shortened to about 6 min during the composting process, and the length of the aeration cycle could also be shortened. The cost of aeration is based on the amount of time during which the blower is operating. Thus, it is possible that controlling a compost pile using oxygen-monitoring equipment could reduce operational costs because the blower may not need to operate as much.

Supplying oxygen, lowering the temperature, and removing moisture from the composting pile are the three main functions of aeration. If one simply considers oxygen supply, it is probable that controlling compost aeration using oxygen levels is better and less costly than the timer/temperature feedback control. Furthermore, controlling oxygen content to a certain range, but not to a high level, may be a critical measure for odor management [18,20].

## 4. Conclusions

The proposed monitoring equipment in this paper can be used to measure oxygen concentration in a composting pile. The test results showed that this system was accurate, stable, and had good responses with good repeatability. The equilibrium time required to detect oxygen concentration in a composting pile was 50 s. Online and real-time oxygen-concentration monitoring was realized with this equipment, and the compost aeration strategy could be automatically optimized depending on the concentration indicated by the monitoring equipment.
